# The Predictive Value of Left Atrial Strain Following Transcatheter Aortic Valve Implantation on Anatomical and Functional Reverse Remodeling in a Multi-Modality Study

**DOI:** 10.3389/fcvm.2022.841658

**Published:** 2022-04-25

**Authors:** Borbála Vattay, Anikó Ilona Nagy, Astrid Apor, Márton Kolossváry, Aristomenis Manouras, Milán Vecsey-Nagy, Levente Molnár, Melinda Boussoussou, Andrea Bartykowszki, Ádám L. Jermendy, Tímea Kováts, Emese Zsarnóczay, Pál Maurovich-Horvat, Béla Merkely, Bálint Szilveszter

**Affiliations:** ^1^Cardiovascular Imaging Research Group, Heart and Vascular Center, Semmelweis University, Budapest, Hungary; ^2^Heart and Vascular Center, Semmelweis University, Budapest, Hungary; ^3^Department of Medicine, Karolinska Institute, Stockholm, Sweden; ^4^Medical Imaging Center, Semmelweis University, Budapest, Hungary

**Keywords:** left atrial function, transcatheter aortic valve implantation, reverse remodeling, CT angiography, speckle tracking echocardiography

## Abstract

**Introduction:**

Transcatheter aortic valve implantation (TAVI) can improve left ventricular (LV) mechanics and survival. Data on the predictive value of left atrial (LA) strain following TAVI are scarce. We aimed to evaluate the association of LA strain measured shortly post-TAVI with functional and anatomical reverse remodeling of the LA and LV, and its association with mortality.

**Methods:**

We prospectively investigated 90 patients who underwent TAVI. Transthoracic echocardiography including strain analysis was performed shortly after TAVI and repeated 6 months later. CT angiography (CTA) was performed for pre-TAVI planning and 6 months post-TAVI. Speckle tracking echocardiography was used to determine LA peak reservoir strain (LASr) and LV global longitudinal strain (LV-GL), LA volume index (LAVi) was measured by TTE. LV mass index (LVMi) was calculated using CTA images. LA reverse remodeling was based on LASr and LAVi changes, whereas LV reverse remodeling was defined as an improvement in LV-GLS or a reduction of LVMi. The association of severely reduced LASr (<20%) at baseline with changes (Δ) in LASr, LAVi, LV-GLS and LVMi were analyzed using linear regression, and Cox proportional hazard model for mortality.

**Results:**

Mean LASr and LV-GLS were 17.7 ± 8.4 and −15.3 ± 3.4% at baseline and 20.2 ± 10.2 and −16.6 ± 4.0% at follow-up (*p* = 0.024 and *p* < 0.001, respectively). Severely reduced LASr at baseline was associated with more pronounced ΔLASr (β = 5.24, *p* = 0.025) and LVMi reduction on follow-up (β = 5.78, *p* = 0.036), however, the majority of the patients had <20% LASr on follow-up (44.4%). Also, ΔLASr was associated with ΔLV-GLS (adjusted β = 2.10, *p* < 0.001). No significant difference in survival was found between patients with baseline severely reduced LASr (<20%) and higher LASr (≥20%) (*p* = 0.054).

**Conclusion:**

LV reverse remodeling based on LVMi was present even in patients with severely reduced LASr following TAVI, although extensive LA damage based on LA strain was demonstrated by its limited improvement over time.

**Clinical Trial Registration:**

(ClinicalTrials.gov number: NCT02826200).

## Introduction

Aortic valve stenosis (AS) is the most common valvular heart disease with growing prevalence in developed countries (1, 2). Symptomatic, severe AS is associated with high morbidity and mortality, therefore early identification of high-risk patients is of major importance. Severe AS induces structural and functional changes of the cardiac chambers due to chronically increased afterload (3). The resulting left ventricular (LV) remodeling with concentric hypertrophy, myocardial fibrosis, and diastolic and systolic dysfunction is associated with poor clinical outcome (4). The left atrium (LA) is also affected by AS leading to LA enlargement, fibrosis, elevated stiffness and reduced LA contractility, increasing the risk of atrial fibrillation and heart failure (5–7). Transcatheter aortic valve implantation (TAVI) is a safe and effective treatment for severe and symptomatic AS patients as an alternative to surgical aortic valve replacement (8, 9). Consequently, LV afterload rapidly decreases resulting in LV reverse remodeling, which is linked to improved cardiac mechanics, symptoms and mortality (10). However, it is unclear whether similar alterations are also true regarding LA function.

Myocardial deformation imaging by two-dimensional (2D) speckle-tracking echocardiography (STE) is a robust and reliable tool for the assessment of subclinical and overt myocardial dysfunction (11, 12). LA reservoir strain (LASr) is considered a surrogate of LV filling pressure and allows for the early detection of diastolic dysfunction (13). LASr is strongly associated with the extent of LA fibrosis (14) and is significantly reduced in patients with AS (5). LA functional deterioration might precede LA dilatation, therefore the assessment of LASr might have incremental value over conventional echocardiographic parameters in patients with AS (15, 16). Moreover, decreased LASr has been shown to have a strong predictive value for various cardiovascular events (17, 18). Incorporating LASr as part of the clinical assessment of TAVI patients could provide useful information for risk stratification and treatment optimization.

Data on the prognostic value of LA strain assessed after TAVI before hospital discharge are scarce. Therefore, in this multimodality study, we sought to assess the influence of LASr measured shortly post-TAVI on both functional and anatomical reverse remodeling of the LA and the LV. Furthermore, we aimed to assess the prognostic value of LASr on all-cause mortality.

## Materials and Methods

### Study Population

The RETORIC (Rule out Transcatheter Aortic Valve Thrombosis with Post Implantation Computed Tomography) trial (NCT02826200) aimed to determine the predictors, incidence and clinical relevance of subclinical leaflet thrombosis in patients with severe, symptomatic AS following TAVI. We prospectively enrolled a total of 154 patients who underwent multimodality imaging (serial CTA, brain MRI and echocardiography) as part of the trial and clinical data were collected in a central database at a tertiary academic center. The current investigation is a sub-study of the RETORIC trial focusing on the clinical value of global strain parameters.

The imaging protocol included transthoracic echocardiography (TTE) including strain analysis to assess LA and LV anatomy and function immediately after (baseline) and 6 months (follow-up) following TAVI. Patients also had CT angiography (CTA) prior to and 6 months after the procedure. Follow-up CTA scans were performed on the same day as the follow-up TTE. Serial CTA images were used to determine LV mass (LVM) changes due to the excellent reproducibility and well-established correlation with gold standard magnetic resonance imaging (19).

**Figure 1:**
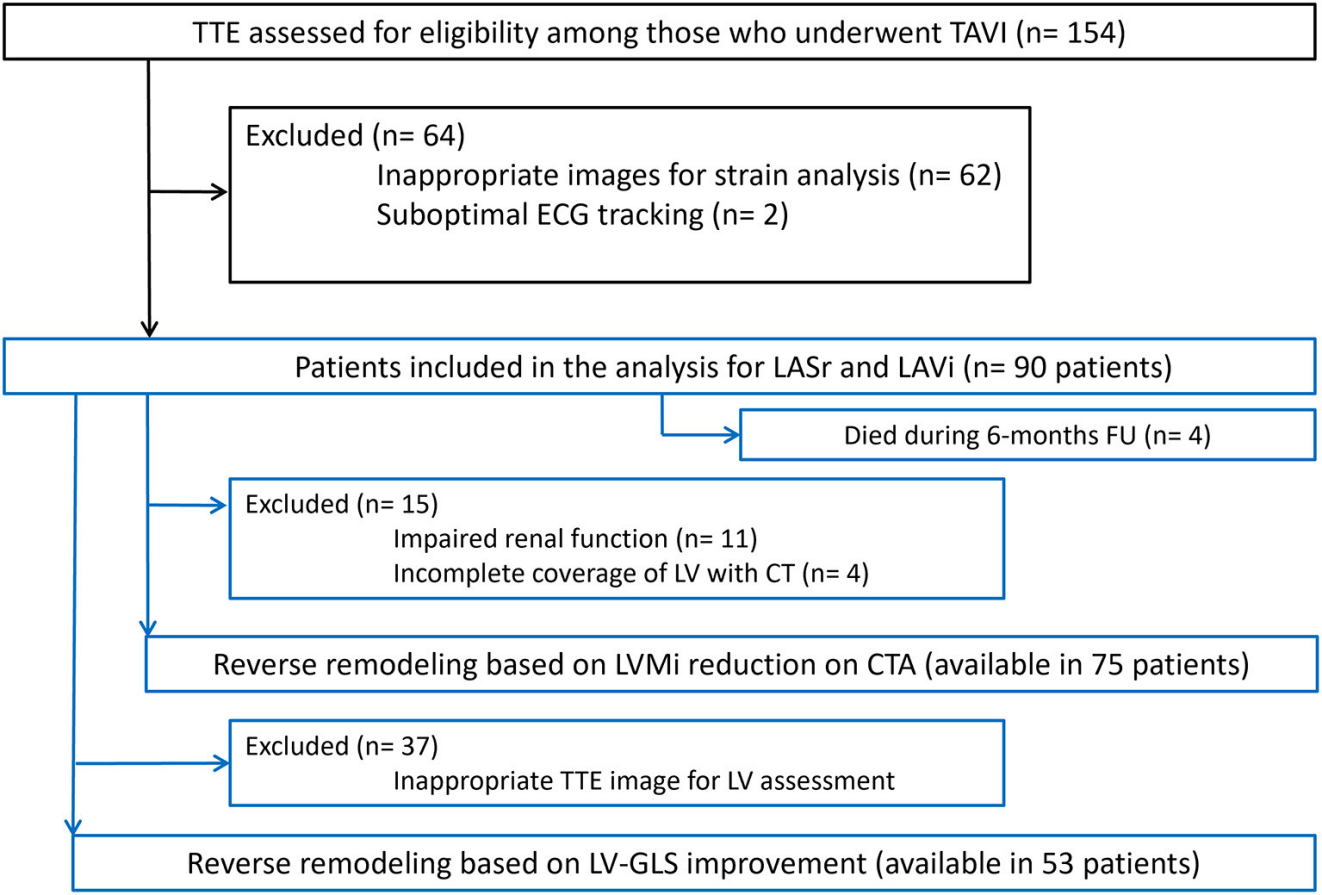
Flowchart of patients through the study. CTA, CT angiography; ECG, Electrocardiography; LA, Left atrium; LASr, Left atrial peak reservoir strain; LAVi, Left atrial volume index; LV-GLS, Left ventricular global longitudinal strain; LVMi, Left ventricular mass index; TAVI, Transcatheter aortic valve implantation; TTE, Transthoracic echocardiography.

Patients were excluded if the quality of echocardiographic images was inadequate for strain analysis, or if CTA was contraindicated per institutional standard of care (inability to cooperate with scan acquisition and/or breath-hold instructions, history of severe and/or anaphylactic contrast reaction, severe renal insufficiency defined as GFR ≤ 30 ml/min/1.73 m^2^) (Figure 1).

The study was approved by the local and national ethical committees (IRB: SE TUKEB 234/2017; ENKK 034489-004/2016/OTIG) and was registered on the ClinicalTrials.gov (NCT02826200) and was performed in accordance with the Helsinki declaration. Written informed consent was obtained from all patients.

### Echocardiographic Analysis

All patients underwent echocardiography before hospital discharge after TAVI and at the 6-month follow-up according to current recommendations, using an EPIQ 7C system (Philips Medical System, Andover, MA), equipped with an X5-1 matrix transducer. 2D gray-scale images (frame rate: 60–80 Hz) were acquired over 3 heart cycles, and stored and analyzed off-line using QLab software (version 15.0 Philips Medical System, Andover, MA) by a single experienced echocardiographer, blinded to clinical and CTA imaging data. Volumetric analysis of the LA and LV were performed using standard methodology recommended by the current chamber quantification guidelines (20). The 2016 guideline document on echocardiographic assessment of diastolic function was utilized to determine filling pressures using the recommended algorithm for patients with preserved and reduced ejection fraction based on the following parameters: LAVi, E/A ratio, E/e′ ratio, e' velocity and peak velocity of the tricuspid regurgitant jet (21). LAVi was determined using biplane Simpson method. Elevated filling pressure was defined as diastolic dysfunction grade 2 and 3 according to the above referenced guideline algorithm. To calculate E/e', both the lateral and septal early diastolic peak longitudinal mitral annular velocities (e′) were obtained and averaged. Pulmonary artery systolic pressure (PASP) was derived from the simplified Bernoulli equation from the peak tricuspid regurgitant velocity and the addition of an estimated value of right atrial pressure.

For LASr and LV-GLS measurements, 2D speckle tracking software (Qlab15 AutoStrain LA for LASr and aCMQ for LV-GLS) were used. Both packages provide automatic endocardial border recognition, that was corrected manually, if needed. LASr was measured in the apical 4-chamber view, the reference point for zero strain was set at LV end-diastole (i.e. beginning of the QRS complex) as recommended by the EACVI/ASE/ Industry Task Force to standardize deformation imaging (22). Severely reduced LASr was defined as <20% and was used in the statistical methods as a categorical variable (23). LA stiffness is a further parameter of atrial function reflecting the change in pressure required to increase the volume of LA in a given measure. It was calculated as ratio of E/e′ to LASr (24). LV-GLS was derived from the apical 4- and 2-chamber views.

### CT Image Acquisition and Analysis

All patients underwent contrast enhanced, multi-detector CT scans using a 256-slice CT scanner (Brilliance iCT 256, Philips Healthcare, Best, The Netherlands, 270 msec rotation time, tube voltage 100–120 kV depending on body weight). For TAVI planning, retrospectively gated helical CTA of the aorta and the heart was acquired during a single breath-hold, in cranio-caudal direction with 1 mm slice thickness and 1 mm increment. We administered 75 ml 400 mg/ml iodinated contrast agent (Iomeron 400, Bracco Ltd; Milan, Italy) with 4.5 ml/s flow rate. Follow-up CT imaging was performed using retrospective gating with a reduced scan range covering the volume of the heart and the ascending aorta. All axial images were reconstructed with 1 mm slice thickness using iterative reconstruction (iDose4 Philips Healthcare, Cleveland, OH, USA). We measured LVM on pre-TAVI and follow-up CTA images in end-systolic or end-diastolic phases (25) using a semi-automated software (Philips Intellispace v6.0.4., Functional analysis tool, Best, The Netherlands). After automatic segmentation of the heart, a single reader manually corrected the LV boundaries excluding papillary muscles on short-axis stack if needed (on 9 slices with 3 mm slice thickness). Papillary muscles were excluded from LVM measurements. Then the software automatically calculated myocardial mass based on volumetric analysis of the LV. LVM values were indexed to the patients' body surface area.

### Evaluation of Reverse Remodeling

Functional LA reverse remodeling was defined as any improvement in LASr, while anatomical LA reverse remodeling was defined as any reduction in LAVi during follow-up. We defined functional LV reverse remodeling as any improvement in LV-GLS on follow-up compared to baseline, whereas anatomical LV reverse remodeling as a reduction in LVMi assessed on follow-up CT images compared to pre-TAVI CT scans. Predictors of LA and LV changes (ΔLASr, ΔLAVi, ΔLV-GLS, ΔLVMi) were analyzed.

### Statistical Analysis

Continuous variables are presented as mean and standard deviation, whereas categorical parameters are presented as frequencies with percentages. Continuous clinical and imaging variables between baseline and follow-up were compared using paired *t*-test or were compared between groups by the two-sample *t*-test (patients with severely reduced LASr (<20%) vs. patients with LASr ≥20%) (Supplementary Table 1). Categorical variables were compared using the Chi-Square test. Based on former studies, we evaluated baseline LASr as a categorical variable in the statistical models as a predictor with a cut-off value defined as <20% (23). However, we have also calculated the models using LASr as continuous variable as seen in Supplementary Tables 2, 3. Relevant imaging parameters (including baseline LASr <20%) and comorbidities as predictors of LA and LV functional (LASr and LV-GLS) and anatomical (LAVi and LVMi) reverse remodeling were analyzed using linear regression analysis. Hypertension was defined as systolic blood pressure >140 mmHg and/or diastolic blood pressure >90 mmHg or antihypertensive medication use verified by medical records. Variables with *p* < 0.05 in univariate analysis were entered into the multivariate model. Spearman correlation was used to assess the relationship between LA and LV parameters. Intraclass correlation coefficient (ICC) was calculated to assess intrareader reproducibility for LASr measurements including 15 patients.

Mortality data were collected and verified via official death records of the National Health Insurance Fund that ensured that no lost-to follow-up occurred. All-cause mortality was used to generate Kaplan–Meier curves for patients with more and <20% LASr at baseline. All analyses were conducted using STATA v13.0. A two-sided *p*-value < 0.05 was considered statistically significant.

## Results

In total, we enrolled 90 patients in our analysis, who had appropriate baseline echocardiography images for LASr measurement (mean age 78.5 ± 6.9 years, mean body mass index (BMI) 28.1 ± 5.6 kg/m^2^, 46.7% female). Four patients died during the first 6 months of follow-up who had baseline LASr assessment. Serial CTA images for LVMi change measurement were available in 75 out of the 90 (83.3%) cases, while 53 of 90 (59.0%) patients also had proper echocardiography images for LV-GLS evaluation (see Figure 1). Patient characteristics, medical treatment and valve types are summarized in Table 1. We have found excellent intrareader reproducibility for LASr measurements including 15 patients with ICC = 0.97.

**Table 1:** Patient characteristics.

**Patient data**	** <20% LASr at baseline** **(*N* = 60)**	**≥20% LASr at baseline** **(*N* = 30)**	***p*-value**
Male, *n* (%)	30 (50.0)	18 (60.0)	0.502
Age, years	79.1 ± 6.7	77.4 ± 7.4	0.383
**Medical history**			
BMI, kg/m^2^	28.3 ± 5.9	27.7 ± 5.2	0.778
Hypertension, *n* (%)	55 (91.7)	26 (86.7)	0.474
Diabetes mellitus, *n* (%)	28 (46.7)	9 (30.0)	0.174
Hyperlipidaemia, *n* (%)	44 (73.3)	21 (70.0)	0.805
COPD, *n* (%)	14 (23.3)	6 (20.0)	0.720
History of TIA/stroke, *n* (%)	6 (10.0)	2 (6.7)	0.600
Prior myocardial infarction, *n* (%)	11 (18.3)	7 (23.3)	0.398
PM/ICD/CRT, *n* (%)	7 (11.7)	2 (6.7)	0.731
Prior syncope, *n* (%)	6 (10.0)	7 (23.3)	0.898
Atrial fibrillation, *n* (%)	21 (35.0)	8 (26.7)	0.116
**Medication**			
Oral anticoagulant therapy, *n* (%)	20 (33.3)	5 (16.7)	0.096
Single antiplatelet therapy, *n* (%)	34 (56.7)	12 (40.0)	0.136
Dual antiplatelet therapy, *n* (%)	7 (11.7)	13 (43.3)	<0.001
*Valve type, n (%)*			0.998
Corevalve/Evolut R	48 (80.0)	24 (80.0)	
Portico	12 (20.0)	6 (20.0)	

*BAV, Balloon aortic valvuloplasty; BMI, Body mass index; BSA, Body surface area; COPD, Chronic obstructive pulmonary disease; CRT, Cardiac resynchronization therapy; ICD, Implantable cardioverter-defibrillator; LASr, Left atrial peak reservoir strain; PM, Pacemaker; TIA, Transient ischemic attack Continuous variables are described as mean ± SD, or median and interquartile range, whereas categorical variables are represented as frequencies and percentage*.

### The Impact of TAVI on LA Anatomy and Function

**Figure 2:**
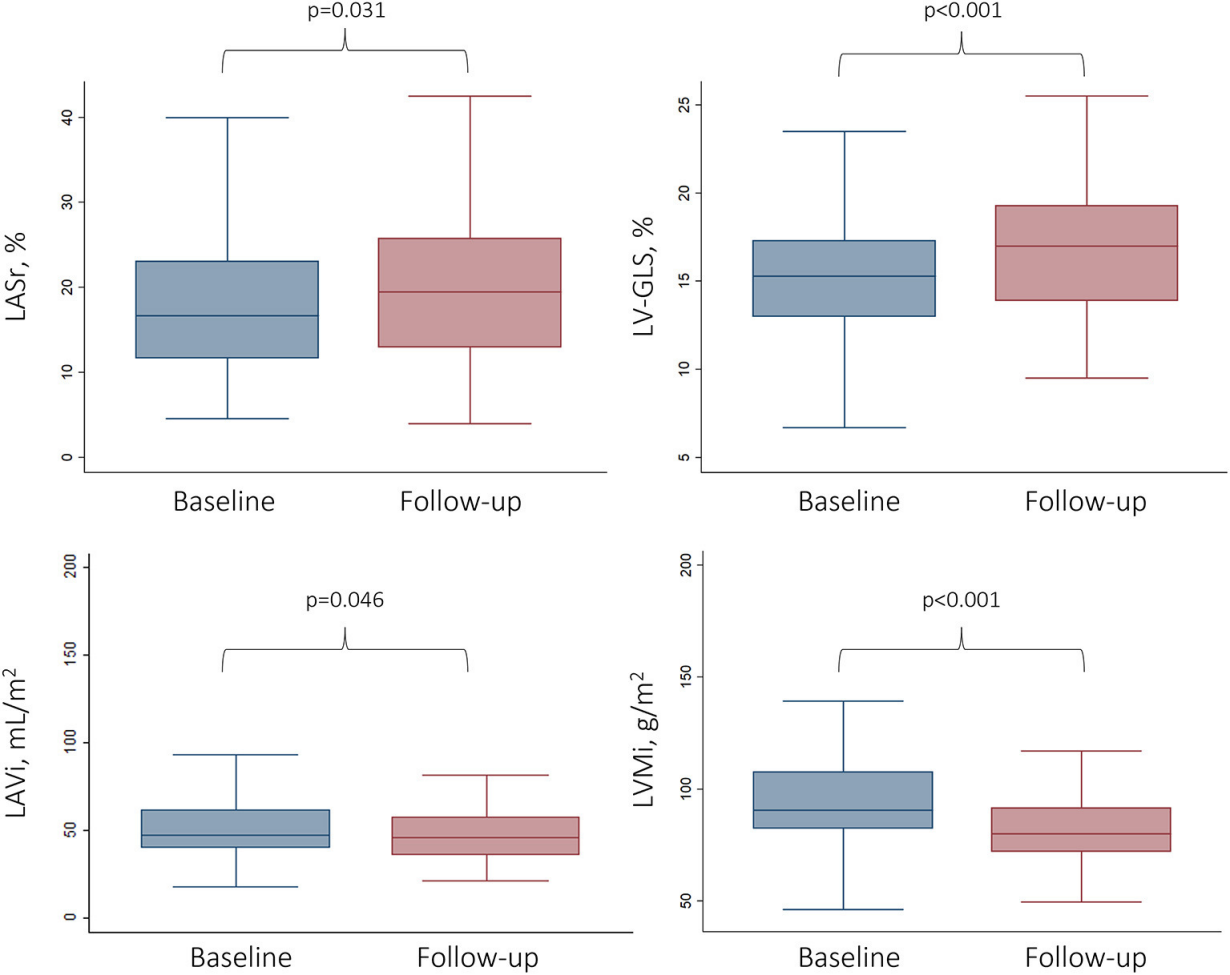
Changes in functional and anatomical LA and LV parameters following TAVI. Box and whisker plots represent the baseline vs. the follow-up values (6 months after TAVI) for LA and LV parameters. LV-GLS, Left ventricular global longitudinal strain; LASr, Left atrial peak reservoir strain; LAVi, Left atrial volume index; LVMi, Left ventricular mass index.

Any improvement in LASr was detected in 60.6% of all patients during follow-up. We found significant improvement comparing baseline and follow-up LASr values (17.7 ± 8.4 vs. 20.2 ± 10.2%, respectively; *p* = 0.024) (Figure 2). There was no significant difference in the proportion of severely reduced LASr (<20%) at baseline and follow-up, respectively [66.7 (60/90) vs. 44.4% (40/90); *p* = 0.134] (Table 2).

**Table 2:** Periprocedural data and change in systolic and diastolic parameters.

**Imaging parameters**	**Baseline**	**6 months follow-up**	**p value**	**Change during follow-up**
LASr, % (*N =* 86)	17.7 ± 8.4	20.2 ± 10.2	0.024	2.4 ± 9.0
LAVi, mL/m^2^ (*N =* 86)	53.8 ± 21.7	50.6 ± 24.9	0.109	−3.2 ± 17.1
LV-GLS, % (*N =* 53)	−15.3 ± 3.4	−16.6 ± 4.0	<0.001	−1.4 ± 2.2
LVMi, g/m^2^ (*N =* 75)	96.2 ± 23.8^*^	83.3 ± 19.5	<0.001	−12.9 ± 13.1
LA stiffness (*N =* 86)	1.2 ± 1.1	1.1 ± 1.1	0.603	−0.1 ± 1.4
Ejection fraction, % (*N =* 86)	55.0 ± 10.2	56.5 ± 8.4	0.060	1.4 ± 6.8
E wave velocity, cm/s (*N =* 86)	99.9 ± 34.4	95.9 ± 32.7	0.288	−4.0 ± 33.8
E/e' (*N =* 85)	16.0 ± 7.1	15.6 ± 6.7	0.651	−0.4 ± 8.3
E/A (*N =* 65)	0.97 ± 0.48	0.88 ± 0.39	0.216	−0.09 ± 0.58
PASP, mmHg (*N =* 74)	41.4 ± 13.9	38.7 ± 12.1	0.175	−2.7 ± 15.6
Mean aortic transvalvular gradient, mmHg (*N =* 86)	9.7 ± 6.4	8.3 ± 6.0	0.014	−1.4 ± 4.8
Paravalvular leak (≥ grade 2), *n* (%)	–	15 (16.7)	–	–
Severely reduced LASr (<20%), *n* (%)	60 (66.7)	40 (44.4)	0.134	–

* *Pre-TAVI*.

*LA, Left atrium; LASr, Left atrial peak reservoir strain; LV, Left ventricle; LV-GLS, Left ventricular global longitudinal strain; LVMi, Left ventricular mass index*.

LASr correlated significantly with diastolic parameters both at baseline (E wave velocity Spearman's ρ = −0.46, LAVi ρ = −0.47, PASP ρ = −0.30; *p* < 0.01 for all) and follow-up (E wave velocity Spearman's ρ = −0.28, LAVi ρ = −0.48, PASP ρ = −0.37; *p* < 0.01 for all). Diastolic parameters differed significantly between both time points (all *p* < 0.05, Table 2). Mean LAVi was 53.8 ± 21.7 mL/m^2^ vs. 50.6 ± 24.9 mL/m^2^ at baseline and follow-up (*p* = 0.109). Baseline LA stiffness was 1.6 ± 1.1 for patients with LASr <20 and 0.5 ± 0.2 for patients with LASr ≥ 20% (*p* < 0.001).

**Figure 3:**
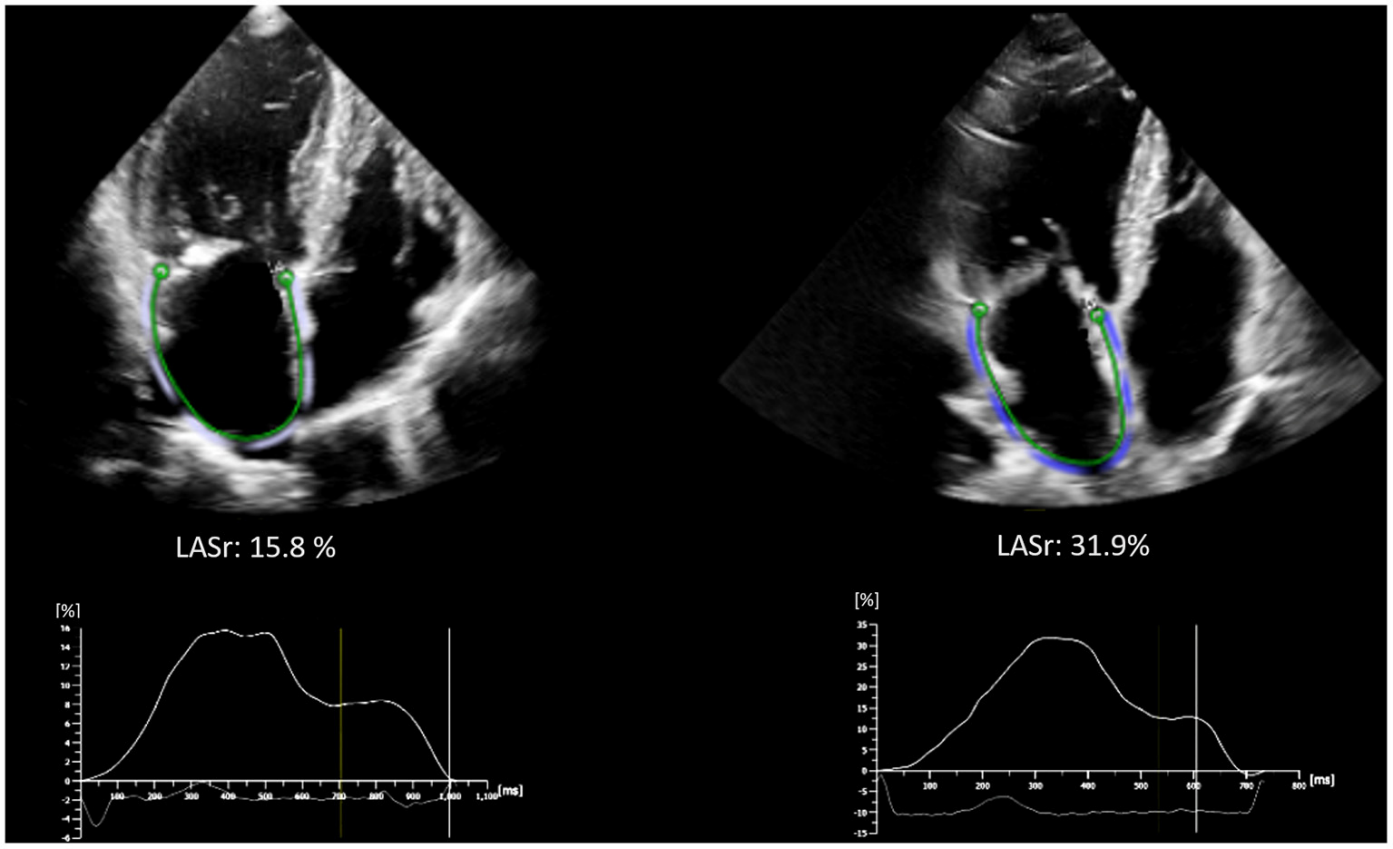
Representative images of LA strain assessment of patients with baseline LASr <20% (left side, LASr = 15.8 %) and ≥ 20% (right side, LASr = 31.9%). Strain curves are depicted below the echocardiographic images. LA, Left atrium; LASr, Left atrial peak reservoir strain.

Among patients with severely reduced LASr (<20%), although some degree of LASr improvement was detectable (mean improvement of 4.4 ± 8.4%), the mean LASr at 6 months was still significantly lower (LASr <20% at baseline: 17.3 ± 9.6% vs. LASr ≥ 20% at baseline: 26.1 ± 9.0%, *p* < 0.001), in turn the LA stiffness was higher as compared to those with LASr ≥ 20% at baseline (1.4 ± 1.2 vs. 0.5 ± 0.4, respectively *p* < 0.001) (see Supplementary Table 1). Representative images of LA strain assessment are shown on Figure 3.

### The Impact of TAVI on LV Anatomy and Function

The LV showed an improvement in both functional and morphological aspects following TAVI: mean LV-GLS was −15.3 ± 3.4% at baseline vs. −16.6 ± 4.0% at follow-up (*p* < 0.001), whereas mean LVMi reduced from 96.2 ± 23.8 g/m^2^ to 83.3 ± 19.5 g/m^2^ on follow-up CTA (*p* < 0.001) (Table 2). Any improvement in LV-GLS was found in 74.5% of all patients during follow-up.

### Predictors of LA Reverse Remodeling

Based on Spearman correlation, ΔLV-GLS was significantly associated with ΔLASr (ρ = 0.594, *p* < 0.001). On multivariate linear regression analysis, ΔLASr was predicted by severely reduced baseline LASr (< 20%) (β = 5.24; *p* = 0.025) and ΔLV-GLS during follow-up (β = 2.10; *p* < 0.001). In a separate model using LASr as a continuous variable, baseline atrial strain was independently associated with ΔLASr (ß = −0.26; *p* = 0.035) (Supplementary Table 2).

LA anatomical reverse remodeling based on LAVi was independently associated with baseline age (β = 0.83; *p* = 0.006) and E/e' ratio (β = −0.86; *p* = 0.001, Table 3), but not with severely impaired LASr at baseline (*p* = 0.396).

**Table 3:** Uni- and multivariate linear regression analysis of the association of comorbidities, echocardiographic parameters and LA reverse remodeling.

	**Univariate**			**Multivariate**		
	**beta**	**95% CI**	***p***	**beta**	**95% CI**	***p***
**Δ** **LASr**						
**Clinical parameters**						
Age (years)	**0.33**	**0.010–0.650**	**0.044**	0.17	−0.179–0.516	0.334
Female sex	−0.67	−4.830–3.492	0.749			
BMI (kg/m^2^)	−0.23	−0.591–0.131	0.209			
Hypertension	−1.25	−8.726–6.233	0.741			
Diabetes mellitus	3.72	−0.416–7.864	0.077			
Atrial fibrillation	−0.72	−3.412–1.969	0.594			
Prior AMI	−4.59	−9.700–0.531	0.078			
**Baseline imaging parameters**						
Elevated LA stiffness	4.58	−0.041–9.198	0.051			
EF (%)	0.17	−0.051–0.388	0.131			
LAVi (mL/m^2^)	−0.06	−0.147–0.036	0.230			
E/e' ratio	0.22	−0.054–0.503	0.112			
Severely reduced LASr (<20%)	**5.58**	**1.337–9.822**	**0.011**	**5.24**	**0.694–9.792**	**0.025**
LVMi (g/m^2^)	−0.02	−0.060–0.027	0.456			
PASP (mmHg)	−0.10	−0.255–0.046	0.171			
LV–GLS (%)	−0.15	−0.876–0.575	0.679			
**Change in imaging parameters**						
Δ LVMi (g/m^2^)	0.00	−0.095–0.095	0.998			
Δ LV–GLS (%)	**2.22**	**1.198–3.246**	**<0.001**	**2.10**	**1.117–3.075**	**<0.001**
**Δ** **LAVi**						
**Clinical parameters**						
Age (years)	**0.67**	**0.057–1.274**	**0.032**	**0.83**	**0.244–1.408**	**0.006**
Female sex	2.27	−5.578–10.123	0.566			
BMI (kg/m^2^)	−0.40	−1.080–0.291	0.255			
Hypertension	0.3	−12.675–12.933	0.984			
Diabetes mellitus	−0.76	−8.652–7.134	0.849			
Atrial fibrillation	−5.60	−13.974–2.780	0.187			
**Baseline imaging parameters**						
Elevated LA stiffness	−7.11	−15.795–1.586	0.108			
EF (%)	0.06	−0.326–0.435	0.775			
LAVi (mL/m^2^)	−0.16	−0.333–0.022	0.084			
E/e' ratio	**−0.74**	**−1.273-−0.209**	**0.007**	**−0.86**	**−1.377–−0.346**	**0.001**
Severely reduced LASr (<20%)	−3.60	−11.993–4.795	0.396			
LVMi (g/m^2^)	−0.05	−0.131–0.034	0.247			
PASP (mmHg)	−0.29	−0.625–0.054	0.098			
LV–GLS (%)	−0.02	−1.488–1.454	0.982			
**Change in imaging parameters**						
Δ LV–GLS (%)	−0.37	−2.823–2.075	0.760			
Δ LASr (%)	−0.20	−0.709–0.300	0.422			
Δ LVMi (g/m^2^)	−0.29	−0.589–0.009	0.057			

*Variables with p < 0.05 in univariate analysis were entered into the multivariate model*.

*AMI, Acute myocardial infarction; BMI, Body mass index; EF, Ejection fraction; LA, Left atrium; LASr, Left atrial peak reservoir strain; LAVi, Left atrial volume index; LV, Left ventricle; LV–GLS, Left ventricular global longitudinal strain; LVMi, Left ventricular mass index; PASP, Pulmonary artery systolic pressure. Numbers marked in bold are significant predictors of the outcome*.

### Predictors of LV Reverse Remodeling

When assessing the predictors of functional LV reverse remodeling, we found that only ΔLASr was linked to ΔLV-GLS values (β = 0.13; *p* < 0.001) and both parameters improved over time (Table 4). Severely reduced LASr (<20%) (β = 5.78; *p* = 0.036) and LVMi (β = 0.12; *p* < 0.001) measured at baseline were independent predictors of anatomical LV reverse remodeling based on ΔLVMi (Table 4). Regarding the magnitude of change in LVMi, patients with severely reduced LASr (<20%) at baseline demonstrated larger anatomical LV reverse remodeling based on LVMi as compared to patients with higher LASr (≥20%): −15.7 ± 12.2 g/m^2^ vs. −8.3 ± 13.4 g/m^2^; *p* = 0.022 (see Supplementary Table 1). Similar results for anatomical LV reverse remodeling were not detected using baseline LASr as a continuous predictor (see Supplementary Table 3).

**Table 4:** Uni- and multivariate linear regression analysis of the association of comorbidities, echocardiographic parameters and LV reverse remodeling.

	**Univariate**			**Multivariate**		
	**beta**	**95% CI**	***p***	**beta**	**95% CI**	***p***
**Δ** **LV–GLS**						
**Clinical parameters**						
Age (years)	0.02	−0.082–0.119	0.712			
Female sex	0.63	−0.641–1.894	0.325			
BMI (kg/m^2^)	−0.10	−0.212–0.008	0.069			
Hypertension	−1.91	−4.215–0.395	0.102			
Atrial fibrillation	−0.53	−1.319–0.256	0.181			
Prior AMI	−0.81	−2.344–0.718	0.291			
**Baseline imaging parameters**						
Elevated LA stiffness	−0.35	−1.741–1.048	0.620			
EF (%)	0.03	−0.041–0.096	0.430			
LAVi (mL/m^2^)	−0.03	−0.062–0.010	0.154			
E/e' ratio	0.00	−0.078–0.085	0.933			
Severely reduced LASr (<20%)	0.41	−0.907–1.717	0.538			
LVMi (g/m^2^)	−0.01	−0.025–0.001	0.078			
LV–GLS (%)	−0.03	−0.217–0.165	0.786			
**Change in imaging parameters**						
Δ LVMi (g/m^2^)	0.01	−0.020–0.038	0.517			
Δ LASr (%)	**0.13**	**0.069–0.184**	**<0.001**			
**Δ** **LVMi**						
**Clinical parameters**						
Age (years)	0.17	−0.304–0.646	0.475			
Female sex	0.52	−5.677–6.716	0.868			
BMI (kg/m^2^)	0.04	−0.500–0.585	0.876			
Hypertension	2.32	−7.022–11.657	0.622			
Prior AMI	−5.05	−12.387–2.278	0.174			
**Baseline imaging parameters**						
Elevated LA stiffness	−0.03	−7.707–7.639	0.993			
EF (%)	0.11	−0.183–0.399	0.461			
LAVi (mL/m^2^)	0.03	−0.118–0.170	0.718			
E/e' ratio	0.33	−0.116–0.780	0.144			
Severely reduced LASr (<20%)	**7.37**	**1.306–13.432**	**0.018**	**5.78**	**0.397–11.166**	**0.036**
LVMi (g/m^2^)	**0.13**	**0.077–0.185**	**<0.001**	**0.12**	**0.071–0.177**	**<0.001**
LV–GLS (%)	1.05	−0.007–2.106	0.051			
**Change in imaging parameters**						
Δ LV–GLS (%)	−0.35	−2,134–1.435	0.695			
Δ LASr (%)	0.04	−0.346–0.421	0.844			

*Variables with p < 0.05 in univariate analysis were entered into the multivariate model*.

*AMI, Acute myocardial infarction; BMI, Body mass index; EF, Ejection fraction; LA, Left atrium; LASr, Left atrial peak reservoir strain; LAVi, Left atrial volume index; LV, Left ventricle; LV–GLS, Left ventricular global longitudinal strain; LVMi, Left ventricular mass index. Numbers marked in bold are significant predictors of the outcome*.

### Outcome Analysis of All-Cause Mortality Following TAVI

**Figure 4:**
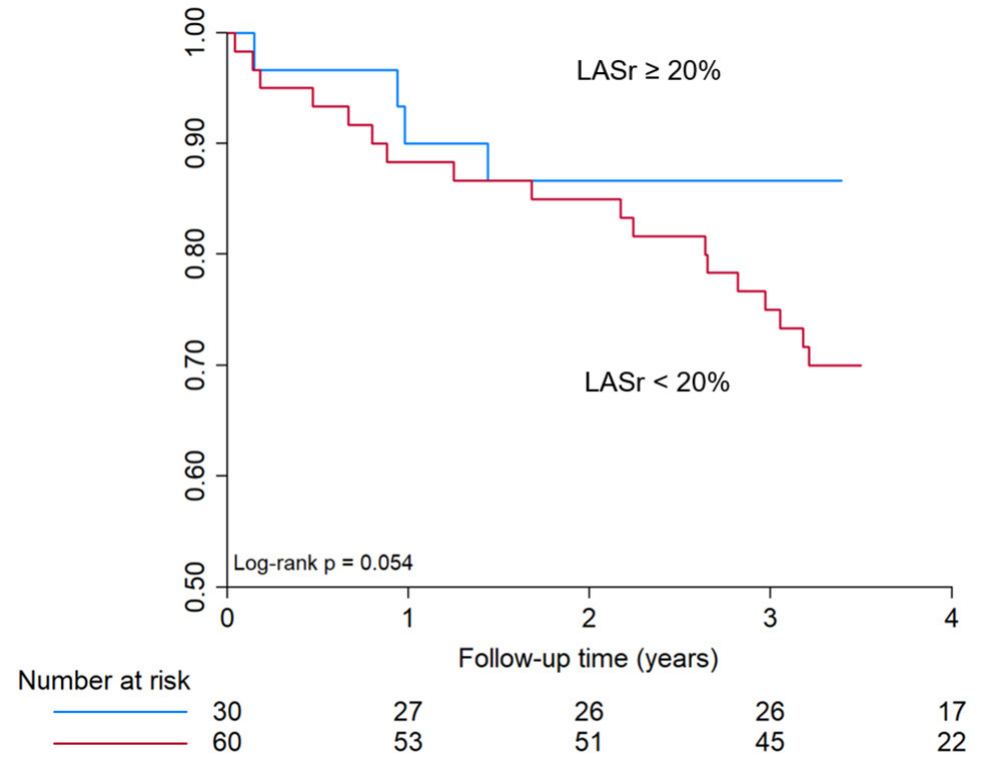
Kaplan-Meier curve for all-cause mortality in patients with <20% or ≥ 20% LASr. Kaplan–Meier curve shows only a trend toward reduced survival in patients with severely reduced LASr at baseline (<20%), however no significant difference was observed during a mean follow-up time of 3.4 ± 1.3 years. LASr, Left atrial peak reservoir strain.

Regarding survival, no significant difference was observed in patients with severely reduced LASr (<20%) as compared to those with higher LASr (≥20%) as assessed immediately after TAVI (log-rank *p*-value 0.054, Figure 4). LASr is a parameter of atrial function that showed interaction with mortality. Baseline comorbidities (hypertension, diabetes mellitus, prior stroke, smoking, age, BMI, atrial fibrillation), parameters of ventricular function, valve type, LVMi or LAVi showed no association with mortality (all *p* > 0.05).

## Discussion

**Figure 5:**
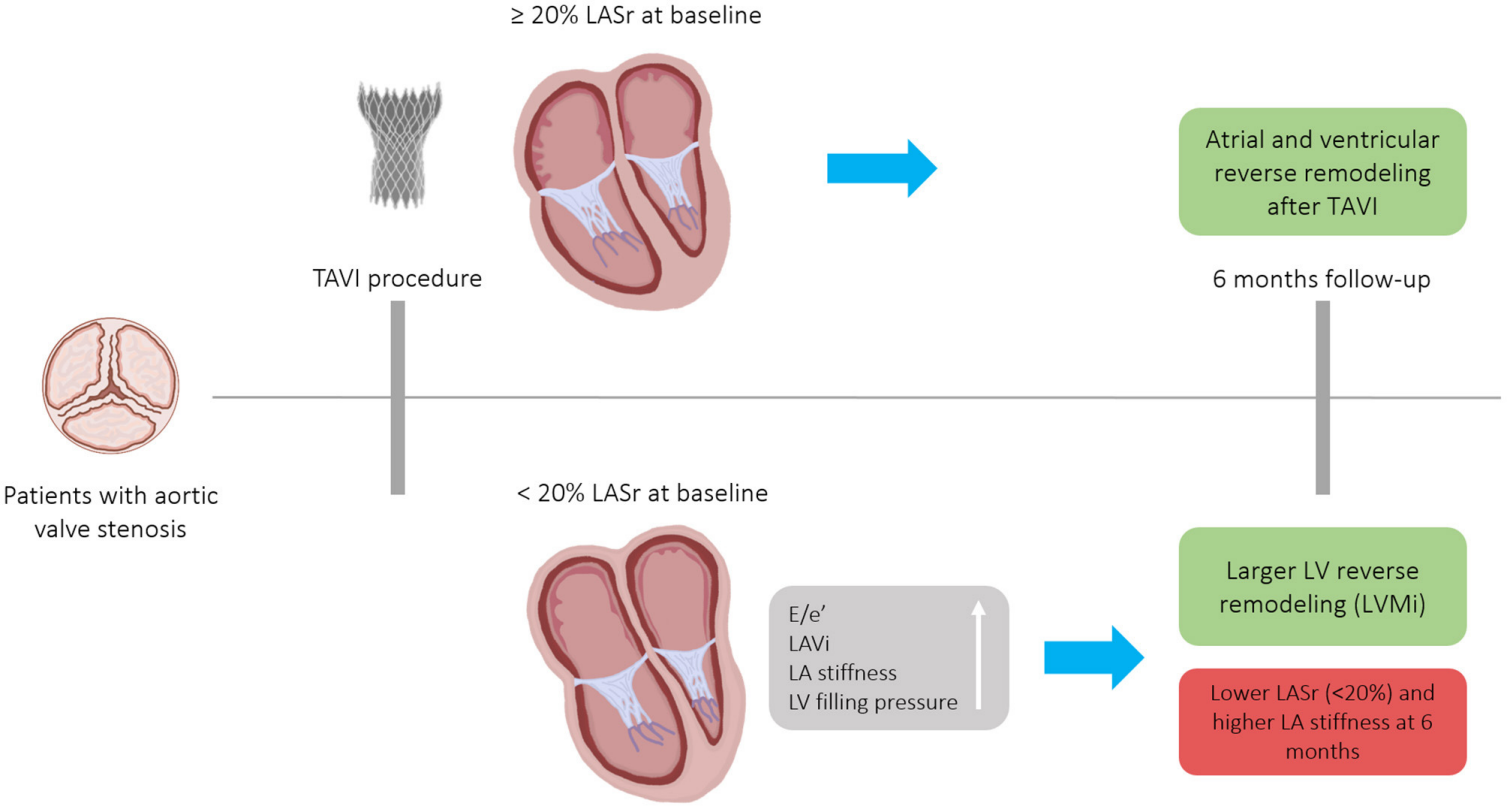
Patients with severe, symptomatic aortic valve stenosis who underwent TAVI experienced atrial and ventricular reverse remodeling after 6-months. The magnitude of anatomical LV and functional LA reverse remodeling was numerically larger in patients with severely reduced baseline LASr (<20%), nonetheless, due to the extensive baseline LA remodeling, these patients remained in the severely reduced LASr range 6 months after a successful TAVI. Patients with LASr <20% had stiffer LA at both time points. E/e', ratio between early mitral inflow velocity and mitral annular early diastolic velocity; LA, Left atrium; LASr, Left atrial peak reservoir strain; LAVi, Left atrial volume index; LV, Left ventricle; TAVI, Transcatheter aortic valve implantation; LVMi, Left ventricular mass index.

In our study, severely reduced LASr at baseline (<20%) was associated with larger change in LASr and LVMi during follow-up, however was not linked to anatomical reverse remodeling of the LA based on LAVi. Notably, majority of the patients with severely reduced LASr at baseline had impaired LA function (LASr <20%) even after 6 months of the procedure despite evidence of some atrial reverse remodeling process (see Figure 5). Both absolute LASr and LV-GLS increased during the follow-up period and the improvement in LA global strain was associated with functional LV reverse remodeling. LASr <20% at baseline was associated with elevated LA stiffness at both time points, although no significant difference regarding survival was observed after TAVI.

Previous studies mainly focused on LV reverse remodeling following surgical aortic valve replacement or TAVI, while less information is available on the anatomical and functional changes of the LA. LA structural remodeling is a dynamic process characterized by chamber enlargement and dysfunction, elevated filling pressure and LA fibrosis. LA remodeling is associated with higher incidence of atrial fibrillation, heart failure hospitalization and death in various patient populations (26–29). LASr is a relatively new and sensitive marker for the evaluation of LA remodeling. Our study is additive to the existing literature, as it highlights the role of baseline LASr with respect to beneficial reverse remodeling of the LA and LV.

The current echocardiographic guideline algorithm uses LAVi in the evaluation of diastolic function (21). However, LASr showed excellent correlation with left ventricular filling pressure independent from LAVi (30), therefore LASr was suggested as a more accurate parameter to assess elevated filling pressure and diastolic dysfunction especially in early phases (13, 31, 32). LA strain can discriminate the severity grade of diastolic dysfunction (33), is associated with higher level of fibrosis of the atrial wall (14) and increased risk for arrhythmias or thromboembolic events (34, 35). Valvular heart disease, sustained arrhythmia, hypertension or heart failure can promote atrial remodeling due to pressure or volume overload leading to changes in form and function (36, 37). Whether this remodeling is reversible is largely unknown. While some studies described an improvement in LA function, a reliable parameter for the assessment of patients with irreversible changes is warranted for proper timing of the procedure. Our study revealed that patients with severe dysfunction immediately after TAVI are predominantly in an advanced stage of atrial remodeling and thus only experience modest improvement in LA strain.

### Functional and Morphological Changes in the LA After TAVI

Former studies reported controversial results regarding atrial reverse remodeling based on functional and morphological LA changes following TAVI. Similarly to our findings, several studies found improvement in LA parameters. Significant improvement was seen in both LA and LV function using TEE in a study comprising 213 patients (38, 39). During a 3 months' follow-up, D'Ascenzi et al. found significant improvement in both functional and anatomical LA parameters: LASr increased from 14.4 ± 3.9 to 19.1 ± 4.7% (*p* < 0.001), LAVi and LA stiffness also decreased significantly (40). In another study, 12 months following TAVI only LASr improved significantly from 21.5 ± 11.2 to 26.6 ± 13.8% (*p* = 0.039), while LAVi remained unchanged (*p* > 0.05) (41). Short-term data by Spethmann et al. also noted an increase in LASr (13.9 ± 5.5 vs. 20.8 ± 8.1%, *p* < 0.001) without the reduction of LAVi (42). This might be explained by recent observations that LA function can improve independently from LA volume. Also, LASr might be a more sensitive parameter for atrial remodeling.

On the other hand, Sabatino et al. did not find any significant improvement in LA morphology or function in 100 patients after TAVI (43). Weber and colleagues evaluated 150 patients and found significant reduction in LAVi by 2.1 ± 10 ml/m^2^, without an increase of LASr, although LA stiffness decreased by 0.38 (*p* = 0.05) (44). In our study, we demonstrate functional, but nomorphological reverse remodeling of the LA 6 months after TAVI. Mean increase in LASr during follow-up was 2.4 ± 9.0% and mean decrease in LAVi was −3.2 ± 17.1 ml/m^2^. Our results suggest that elevated LV filling pressure (based on E/e') hinders anatomical reverse remodeling of the LA based on LAVi change.

Currently there are no studies evaluating LASr improvement following TAVI in respect of LA stiffness. Although LASr improved to some extent (by 4.4 ± 8.4%) during follow-up in patients with severely impaired LA function at baseline, it mostly remained in the severely reduced range (LASr <20%) 6 months after a successful TAVI implantation and LA stiffness also remained significantly elevated. These findings underline the role of early intervention in patients with severe AS before irreversible damage occurs to the atrial tissue. LASr might be an excellent biomarker for early detection of atrial injury and thereby guide patient management and procedure timing.

In our study, improvement in LV functional remodeling was associated with greater improvement in LA function (ρ = 0.594, *p* < 0.001). D'Andrea et al. found that LVMi and LV-GLS measured before TAVI predicted LA functional improvement (β = 0.45 and 0.54, respectively, *p* < 0.001) (39). In other studies, LA volume, mean transvalvular gradient and its change were reported as predictors of LASr increase (40).

Data regarding the predictive value of LASr on cardiovascular outcomes in patients receiving TAVI are limited. The improvement of LASr after TAVI has been reported as an independent predictor of cardiovascular mortality and heart failure hospitalization (43). In previous studies LASr in TAVI patients was also a predictor of new-onset AF after the procedure (44). In our study, survival analysis revealed only a trend toward better survival of patients with baseline LASr ≥ 20%, although the difference was not statistically different.

### LV Reverse Remodeling After TAVI

Beneficial reverse remodeling of the LV after TAVI was predominantly demonstrated by TTE and CTA studies. Our research group previously found that LVM can regress in a magnitude of 43.4 ± 33.9 g during a mean follow-up time of 2.6 years (19). It has also been suggested that reverse remodeling based on LVMi occurs within the first weeks following implantation and is a reliable marker of improved prognosis (45). Interestingly, we found that despite severely reduced LASr values, morphological LV remodeling can occur to a substantial extent. This might be explained by the observation, that even despite long-standing AS, LV can recover following afterload normalization. In our analysis, we detected substantial difference in ΔLVMi when comparing patients with LASr <20 vs. ≥20% but not with LASr as a continuous parameter. Baseline LASr <20% was also linked to improvement in LVMi (ß = 5.78, *p* = 0.036) after adjustment for the baseline LVMi.

Also, in line with prior investigations we found that LV function described by LV-GLS improves after TAVI based on TTE or CT imaging. Echocardiographic data by Alenezi et al. found significant increase in the absolute value of LV-GLS (46). Also, Marwan et al. who measured LV-GLS using CT images reported the recovery of the LV (47). Shiino and colleagues demonstrated that LV-GLS improvement occurs already within 1 month after TAVI (48).

Predictors of LV-GLS improvement in other studies were pre-TAVI LVM, LV-GLS, glomerular filtration rate, diastolic blood pressure and post-TAVI peak creatine kinase-MB level after the procedure (39, 46). Interestingly, patients with normal LV-GLS before TAVI had lower improvement in LV-GLS during follow-up based on the results of Alenezi et al. (46). Based on our data, only improvement in LASr correlated with the change of LV-GLS.

We acknowledge the limitations of our study. After excluding non-diagnostic images, we could assess LVMi and strain parameters in only a subset of patients. This also highlights the limitations of current non-invasive imaging modalities for the assessment of TAVI patients. Data regarding the exact duration of AS before the procedure were not available. All-cause mortality was used for outcome analysis. Former studies included LA reservoir strain as a marker of diastolic assessment as a binary variable with the cut-off value used in current study (20%) that has been also widely used in the clinical setting for the evaluation of LA function. Using LASr as a continuous predictor was associated with ΔLASr but not with ΔLVMi (Supplementary Tables 2, 3).

## Conclusion

LV reverse remodeling based on LVMi occurred even in patients with severely reduced LASr following TAVI, although extensive LA damage was demonstrated in this cohort by limited improvement in LA function over time. During the 6 months follow-up, simultaneous improvement of LV and LA strain was observed. No significant difference was observed in patients with severely reduced LASr (<20%) as compared to those with higher LASr (≥20%).

## Data Availability Statement

The original contributions presented in the study are included in the article/Supplementary Material, further inquiries can be directed to the corresponding author.

## Ethics Statement

The studies involving human participants were reviewed and approved by Local and National Ethical Committee (IRB: SE TUKEB 234/2017; ENKK 034489-004/2016/OTIG). The patients/participants provided their written informed consent to participate in this study.

## Author Contributions

BV, EZ, AN, MB, MV-N, and BS performed image analysis/measurements. MK and BS performed statistical analysis. BV, AN, MK, AA, AM, and BS wrote the manuscript with input from all authors. BM and PM-H contributed to conception and design of the study. AB, TK, MV-N, LM, and ÁJ aided in interpreting the results and worked on the manuscript. LM and BM performed the interventions. All authors contributed to manuscript revision, read, and approved the submitted version.

## Funding

This study was supported by the National Research, Development and Innovation Office of Hungary (NKFIA; NVKP_16-1-2016-0017 National Heart Program). The research was supported by the Thematic Excellence Programme (Tématerületi Kiválósági Program, 2020-4.1.1.-TKP2020) of the Ministry for Innovation and Technology in Hungary, within the framework of the Therapeutic Development and Bioimaging programmes of the Semmelweis University. BS was supported by the ÚNKP-20-4-II New National Excellence Program of the Ministry for Innovation and Technology from the source of the National research, Development and Innovation fund.

## Conflict of Interest

The authors declare that the research was conducted in the absence of any commercial or financial relationships that could be construed as a potential conflict of interest.

## Publisher's Note

All claims expressed in this article are solely those of the authors and do not necessarily represent those of their affiliated organizations, or those of the publisher, the editors and the reviewers. Any product that may be evaluated in this article, or claim that may be made by its manufacturer, is not guaranteed or endorsed by the publisher.
