# Supplementary material for: The Predictive Value of Left Atrial Strain Following Transcatheter Aortic Valve Implantation on Anatomical and Functional Reverse Remodeling in a Multi-Modality Study
Source: Front Cardiovasc Med. 2022 Apr 25;9:841658. doi: 10.3389/fcvm.2022.841658 (PMC9081648; doi:10.3389/fcvm.2022.841658)
Supplement: Supplementary file 1 [file Table_1.DOCX]

**Table S1.** Comparison of patients based on LA function at baseline.

|  | <20% LASr at baseline (N=60) | ≥20% LASr at baseline (N=30) | p value |
| --- | --- | --- | --- |
| Male, n (%) | 30 (50.0) | 18 (60.0) | 0.502 |
| Age, years | 79.1 ± 6.7 | 77.4 ± 7.4 | 0.383 |
| *Medical history* |  |  |  |
| BMI, kg/m^2^ | 28.3 ± 5.9 | 27.7 ± 5.2 | 0.778 |
| Hypertension, n (%) | 55 (91.7) | 26 (86.7) | 0.474 |
| Diabetes mellitus, n (%) | 28 (46.7) | 9 (30.0) | 0.174 |
| Hyperlipidaemia, n (%) | 44 (73.3) | 21 (70.0) | 0.805 |
| Prior myocardial infarction, n (%) | 11 (18.3) | 7 (23.3) | 0.398 |
| AF, n (%) | 21 (35.0) | 8 (26.7) | 0.116 |
| Prior stroke, n (%) | 4 (6.7) | 0 (0.0) | 0.516 |
| **Echocardiography** |  |  |  |
| Mean transaortic gradient, mmHg (pre-TAVI) | 48.1 ± 16.0 | 51.2 ± 15.2 | 0.258 |
| *Baseline* |  |  |  |
| LV-EF, % | 53.9 ± 10.9 | 57.2 ± 8.2 | 0.181 |
| LV-GLS, % | 13.1 ± 4.3 | 16.3 ± 3.3 | 0.170 |
| E velocity, cm/s | 109.4 ± 37.6 | 78.4 ± 20.9 | **<0.001** |
| A velocity, cm/s | 102.8 ± 30.7 | 98.2 ± 23.0 | 0.585 |
| E/A ratio | - 1. ± 0.6 | 0.9 ± 0.5 | 0.343 |
| Mean e’ velocity, cm/s | 6.5 ± 2.1 | 6.6 ± 1.8 | 0.593 |
| E/e’ ratio | 18.0 ± 8.1 | 12.6 ± 3.9 | **0.001** |
| LA volume index , mL/m^2^ | 57.0 ± 22.7 | 44.7 ± 15.2 | **0.008** |
| TAPSE, mm | 19.9 ± 4.8 | 22.0 ± 4.4 | 0.070 |
| PASP , mmHg | 42.8 ± 14.5 | 34.5 ± 10.5 | **0.030** |
| LASr, % | 13.1 ± 4.3 | 27.1 ± 5.4 | **<0.001** |
| LA stiffness | 1.6 ± 1.1 | 0.5 ± 0.2 | **< 0.001** |
| Elevated filling pressure, n (%) | 42 (70.0) | 12 (40.0) | **0.005** |
| *6 months follow-up* |  |  |  |
| LV-EF, % | 56.2 ± 9.0 | 57.0 ± 7.3 | 0.941 |
| LV-GLS, % | 16.2 ± 16.1 | 17.3 ± 17.4 | 0.381 |
| E velocity, cm/s | 104.7 ± 32.3 | 78.2 ± 26.0 | **<0.001** |
| A velocity, cm/s | 95.4 ± 34.9 | 98.5 ± 26.4 | 0.984 |
| E/A ratio | - 1. ± 0.9 | 0.8 ± 0.3 | **0.011** |
| Mean e’ velocity, cm/s | 6.8 ± 5.2 | 8.3 ± 7.1 | 0.453 |
| E/e’ ratio | 17.0 ± 6.9 | 12.1 ± 5.5 | **0.002** |
| LA volume index, mL/m^2^ | 54.4 ± 24.9 | 45.2 ± 28.0 | **0.003** |
| TAPSE, mm | 21.5 ± 5.8 | 23.8 ± 4.5 | 0.088 |
| PASP, mmHg | 39.6 ±11.6 | 30.6 ± 12.8 | **0.005** |
| LASr, % | 17.3 ± 9.6 | 26.1 ± 9.0 | **<0.001** |
| LA stiffness | 1.4 ± 1.2 | 0.5 ± 0.4 | **<0.001** |
| Mean transaortic gradient, mmHg | 7.2 ± 3.6 | 10.6 ± 8.8 | **0.009** |
| Δ LV-GLS, % | 1.5 ± 2.4 | 1.1 ± 1.9 | 0.447 |
| Δ LASr, % | 4.4 ± 8.4 | -1.0 ± 8.3 | **0.015** |
| **CT Imaging** |  |  |  |
| LVM pre-TAVI, g | 183.1 ± 55.3 | 170.3 ± 38.2 | 0.656 |
| LVM at follow-up, g | 153.9 ± 43.8 | 155.3 ± 39.1 | 0.507 |
| Δ LVMi, g/m^2^ | -15.7 ± 12.2 | -8.3 ± 13.4 | **0.022** |

AF: Atrial fibrillation; PASP: Pulmonary artery systolic pressure; BMI: Body mass index; CT: Computed tomography; EF: Ejection fraction; LA: Left atrium; LASr: Left atrial peak reservoir strain; LV: Left ventricle; LV-GLS: Left ventricular global longitudinal strain; LVM: Left ventricular mass; TAPSE: Tricuspid annular plane systolic excursion
