# Supplementary material for: The Predictive Value of Left Atrial Strain Following Transcatheter Aortic Valve Implantation on Anatomical and Functional Reverse Remodeling in a Multi-Modality Study
Source: Front Cardiovasc Med. 2022 Apr 25;9:841658. doi: 10.3389/fcvm.2022.841658 (PMC9081648; doi:10.3389/fcvm.2022.841658)
Supplement: Supplementary file 2 [file Table_2.DOCX]

| **Table S2.** Uni- and multivariate linear regression analysis of the association of comorbidities, echocardiographic parameters and LA reverse remodeling. | | | | | | |
| --- | --- | --- | --- | --- | --- | --- |
|  | Univariate | | | Multivariate | | |
| **Δ LASr** | beta | 95% CI | p | beta | 95% CI | p |
| *Clinical parameters* |  |  |  |  |  |  |
| **Age (years)** | **0.33** | **0.010 - 0.650** | **0.044** | 0.25 | -0.077 - 0.566 | 0.134 |
| Female sex | -0.67 | -4.830 - 3.492 | 0.749 |  |  |  |
| BMI (kg/m^2^) | -0.23 | -0.591 - 0.131 | 0.209 |  |  |  |
| Hypertension | -1.25 | -8.726 - 6.233 | 0.741 |  |  |  |
| Diabetes mellitus | 3.72 | -0.416 - 7.864 | 0.077 |  |  |  |
| Atrial fibrillation | -0.72 | -3.412 - 1.969 | 0.594 |  |  |  |
| Prior AMI | -4.59 | -9.700 - 0.531 | 0.078 |  |  |  |
| *Baseline imaging parameters* |  |  |  |  |  |  |
| **LASr (%)** | **-0.31** | **-0.547 - -0.070** | **0.012** | **-0.26** | **-0.507 - -0.020** | **0.035** |
| Elevated LA stiffness | 4.58 | -0.041 - 9.198 | 0.051 |  |  |  |
| EF (%) | 0.17 | -0.051 - 0.388 | 0.131 |  |  |  |
| LAVi (mL/m^2^) | -0.06 | -0.147 - 0.036 | 0.230 |  |  |  |
| E/e’ ratio | 0.22 | -0.054 - 0.503 | 0.112 |  |  |  |
| LVMi (g/m^2^) | -0.02 | -0.060 - 0.027 | 0.456 |  |  |  |
| PASP (mmHg) | -0.10 | -0.255 - 0.046 | 0.171 |  |  |  |
| LV-GLS (%) | -0.15 | -0.876 - 0.575 | 0.679 |  |  |  |
|  | Univariate | | | Multivariate | | |
| **Δ LAVi** | beta | 95% CI | p | beta | 95% CI | p |
| *Clinical parameters* |  |  |  |  |  |  |
| **Age (years)** | **0.67** | **0.057-1.274** | **0.032** | **0.83** | **0.244 - 1.408** | **0.006** |
| Female sex | 2.27 | -5.578 - 10.123 | 0.566 |  |  |  |
| BMI (kg/m2) | -0.40 | -1.080 - 0.291 | 0.255 |  |  |  |
| Hypertension | 0.3 | -12.675 - 12.933 | 0.984 |  |  |  |
| Diabetes mellitus | -0.76 | -8.652 - 7.134 | 0.849 |  |  |  |
| Atrial fibrillation | -5.60 | -13.974 - 2.780 | 0.187 |  |  |  |
| *Baseline imaging parameters* |  |  |  |  |  |  |
| LASr (%) | 0.20 | -0.284 - 0.682 | 0.414 |  |  |  |
| Elevated LA stiffness | -7.11 | -15.795 - 1.586 | 0.108 |  |  |  |
| EF (%) | 0.06 | -0.326 - 0.435 | 0.775 |  |  |  |
| LAVi (mL/m^2^) | -0.16 | -0.333 - 0.022 | 0.084 |  |  |  |
| **E/e’ ratio** | **-0.74** | **-1.273 - -0.209** | **0.007** | **-0.86** | **-1.377 - -0.346** | **0.001** |
| LVMi (g/m^2^) | -0.05 | -0.131 - 0.034 | 0.247 |  |  |  |
| PASP (mmHg) | -0.29 | -0.625 - 0.054 | 0.098 |  |  |  |
| LV-GLS (%) | -0.02 | -1.488 - 1.454 | 0.982 |  |  |  |
| Variables with p<0.05 in univariate analysis were entered into the multivariate model.  AMI: Acute myocardial infarction; BMI: Body mass index; EF: Ejection fraction; LA: Left atrium; LASr: Left atrial peak reservoir strain; LAVi: Left atrial volume index; LV: Left ventricle; LV-GLS: Left ventricular global longitudinal strain; LVMi: Left ventricular mass index; PASP: Pulmonary artery systolic pressure | | | | | | |
