# Supplementary material for: The Predictive Value of Left Atrial Strain Following Transcatheter Aortic Valve Implantation on Anatomical and Functional Reverse Remodeling in a Multi-Modality Study
Source: Front Cardiovasc Med. 2022 Apr 25;9:841658. doi: 10.3389/fcvm.2022.841658 (PMC9081648; doi:10.3389/fcvm.2022.841658)
Supplement: Supplementary file 3 [file Table_3.DOCX]

| **Table S3.** Uni- and multivariate linear regression analysis of the association of comorbidities, echocardiographic parameters and LV reverse remodeling. | | | | | | | |  |
| --- | --- | --- | --- | --- | --- | --- | --- | --- |
|  | Univariate | | | Multivariate | | | |  |
| **Δ LV-GLS** | beta | 95% CI | p | beta | 95% CI | p | |  |
| *Clinical parameters* |  |  |  |  |  |  | |  |
| Age (years) | 0.02 | -0.082 - 0.119 | 0.712 |  |  |  | |  |
| Female sex | 0.63 | -0.641 - 1.894 | 0.325 |  |  |  | |  |
| BMI (kg/m^2^) | -0.10 | -0.212 - 0.008 | 0.069 |  |  |  | |  |
| Hypertension | -1.91 | -4.215 - 0.395 | 0.102 |  |  |  | |  |
| Atrial fibrillation | -0.53 | -1.319 - 0.256 | 0.181 |  |  |  | |  |
| Prior AMI | -0.81 | -2.344 - 0.718 | 0.291 |  |  |  | |  |
| *Baseline imaging parameters* |  |  |  |  |  |  | |  |
| LASr (%) | -0.02 | -0.100 - 0.053 | 0.540 |  |  |  | |  |
| Elevated LA stiffness | -0.35 | -1.741 - 1.048 | 0.620 |  |  |  | |  |
| EF (%) | 0.03 | -0.041 - 0.096 | 0.430 |  |  |  | |  |
| LAVi (mL/m^2^) | -0.03 | -0.062 - 0.010 | 0.154 |  |  |  | |  |
| E/e’ ratio | 0.00 | -0.078 - 0.085 | 0.933 |  |  |  | |  |
| LVMi (g/m^2^) | -0.01 | -0.025 - 0.001 | 0.078 |  |  |  | |  |
| LV-GLS (%) | -0.03 | -0.217 - 0.165 | 0.786 |  |  |  | |  |
|  |  | Univariate |  |  | Multivariate |  | |  |
| **Δ LVMi** | beta | 95% CI | p | beta | 95% CI | p | |  |
| *Clinical parameters* |  |  |  |  |  |  | |  |
| Age (years) | 0.17 | -0.304 - 0.646 | 0.475 |  |  |  | |  |
| Female sex | 0.52 | -5.677 - 6.716 | 0.868 |  |  |  | |  |
| BMI (kg/m^2^) | 0.04 | -0.500 - 0.585 | 0.876 |  |  |  | |  |
| Hypertension | 2.32 | -7.022 - 11.657 | 0.622 |  |  |  | |  |
| Prior AMI | -5.05 | -12.387 - 2.278 | 0.174 |  |  | |  | |
| *Baseline imaging parameters* |  |  |  |  |  |  | |  |
| LASr (%) | -0.22 | -0.590 - 0.150 | 0.239 |  |  |  | |  |
| Elevated LA stiffness | -0.03 | -7.707 - 7.639 | 0.993 |  |  |  | |  |
| EF (%) | 0.11 | -0.183 - 0.399 | 0.461 |  |  |  | |  |
| LAVi (mL/m^2^) | 0.03 | -0.118 - 0.170 | 0.718 |  |  |  | |  |
| E/e’ ratio | 0.33 | -0.116 - 0.780 | 0.144 |  |  |  | |  |
| **LVMi (g/m^2^)** | **0.13** | **0.077 - 0.185** | **<0.001** |  |  |  | |  |
| LV-GLS (%) | 1.05 | -0.007 - 2.106 | 0.051 |  |  |  | |  |
| Variables with p<0.05 in univariate analysis were entered into the multivariate model.  AMI: Acute myocardial infarction; BMI: Body mass index; EF: Ejection fraction; LA: Left atrium; LASr: Left atrial peak reservoir strain; LAVi: Left atrial volume index; LV: Left ventricle; LV-GLS: Left ventricular global longitudinal strain; LVMi: Left ventricular mass index | | | | | | | |  |
